# Three‐year follow up of using combination therapy with fresh‐frozen plasma and iron chelation in a patient with acaeruloplasminemia

**DOI:** 10.1002/jmd2.12176

**Published:** 2020-10-26

**Authors:** Andreas Tridimas, Godfrey T. Gillett, Sally Pollard, Nandini Sadasivam, Adrian Williams, Kirsty Mellor, Anthony Catchpole, Karolina M. Stepien

**Affiliations:** ^1^ Department of Clinical Biochemistry and Metabolic Medicine Royal Liverpool Hospital Liverpool UK; ^2^ Laboratory Medicine, Northern General Hospital Sheffield Teaching Hospitals NHS Foundation Trust Sheffield UK; ^3^ Paediatrics Department Bradford Teaching Hospitals NHS Foundation Trust, Bradford Royal Infirmary Bradford UK; ^4^ Red cell and General Haematology Department Manchester Royal Infirmary Manchester UK; ^5^ Haematology Department Bradford Royal Infirmary Bradford UK; ^6^ Clinical Nurse Haemoglobinopathy Bradford Royal Infirmary Bradford UK; ^7^ Scottish Trace Element and Micronutrient Diagnostic and Research Laboratory, Department of Clinical Biochemistry Glasgow Royal Infirmary Glasgow UK; ^8^ Adult Inherited Metabolic Diseases Salford Royal Hospital NHS Trust Salford UK; ^9^ Division of Diabetes, Endocrinology and Gastroenterology University of Manchester Manchester UK

**Keywords:** anaemia, caeruloplasmin, fresh‐frozen plasma, iron chelation

## Abstract

Acaeruloplasminemia is a rare autosomal recessive condition caused by inactivating mutations of the *CP* gene encoding caeruloplasmin (ferroxidase). Caeruloplasmin is a copper‐containing plasma ferroxidase enzyme with a key role in facilitating cellular iron efflux. We describe a case of a patient with acaeruloplasminemia, confirmed by genetic analysis, treated with combination therapy of monthly fresh‐frozen plasma (FFP) or Octaplas and iron chelation over a 3‐year period. This 19‐year‐old male was diagnosed at the age of 14 after developing issues with social interaction at school prompting investigation. Prior to this, he had been well with a normal childhood. He was found to have an iron deficient picture with a paradoxically high ferritin, with low serum copper and undetectable caeruloplasmin. Genetic testing identified a homozygous splicing mutation, c.(1713 + delG);(c.1713 + delG), in intron 9 of the caeruloplasmin gene. Ferriscan showed a high liver iron concentration of 5.3 mg/g dry tissue (0.17‐1.8). Brain and cardiac T2‐weighted magnetic resonance (MR) imaging did not detect iron deposition of the brain or heart respectively. Treatment with monthly Octaplas infusion was commenced alongside deferasirox (540 mg o.d.) in an attempt to increase caeruloplasmin levels and reduce iron overload, respectively. After 3 years of treatment, there was biochemical improvement with a reduction in ferritin from 1084 (12‐250) to 457 μg/L, ALT from 87 (<50) to 34 U/L together with improvement in his microcytic anaemia. No significant adverse events occurred. This case report adds further evidence of treatment efficacy and safety of combined FFP and iron chelation therapy in acaeruloplasminemia.

## 1 INTRODUCTION

Acaeruloplasminemia (ACP) is a rare, autosomal recessive condition of adult onset, caused by inactivating mutations affecting the *CP* gene encoding caeruloplasmin (ferroxidase). CP is a copper‐containing ferroxidase enzyme which has a crucial role in cellular iron homeostasis. It is the disruption of CP ferroxidase activity that leads to systemic iron accumulation which over time can result in clinical manifestations such as diabetes mellitus, progressive neurodegeneration, retinopathy, microcytic anaemia and liver disease.^1^ CP is thought to play a key role in protecting the brain from systemic iron deficiency and oxidative damage due to iron overload. Under normal circumstances, serum CP does not cross the blood‐brain barrier. In the brain, most of the CP is located on the surface of astrocytes in a glycosylphosphatidylinositol (GPI)‐anchored form.^2^

In ACP the loss of this important regulatory function is what ultimately results in neurodegeneration as well as causing iron‐mediated damage to other organs such as the liver, pancreas and retina. ACP is the only known iron overload disorder in which both systemic and brain iron overload are present.^1^ Biochemically an “atypical microcytic anaemia” picture of low transferrin saturation, mild microcytic anaemia and hyperferritinaemia is often the first detectable sign of ACP; atypical in the sense that typically ferritin is low in cases of iron deficiency anaemia.^3^

## 2 CASE REPORT

A 12‐year‐old male was referred to a community paediatrician after finding interaction with his peers difficult. Prior to this, he had a normal childhood with no significant illness being reported and no delay in the onset of puberty. He was born at 38 weeks of gestation due to maternal gestational cholestasis. Clinical examination was unremarkable apart from the presence of marked leukonychia. As part of his behavioral workup, he went on to have basic biochemical and hematological testing which identified a microcytic anaemia with a haemoglobin of 10.4 g/L (11.5‐15.0) and mean cell volume of 61 fL (78‐100). Serum iron was low at 6 μmol/L (14‐31) with a total iron‐binding capacity of 67 μmol/L (54‐80), iron‐binding saturation of 6% (20‐55) and an elevated ferritin of 534 μg/L (10‐322). His haemoglobin electrophoresis and haemoglobinopathy genetic test were negative. Further evaluation under pediatrics led to copper assessment, prompted by a raised alanine aminotransferase (ALT).

**FIGURE 1:**
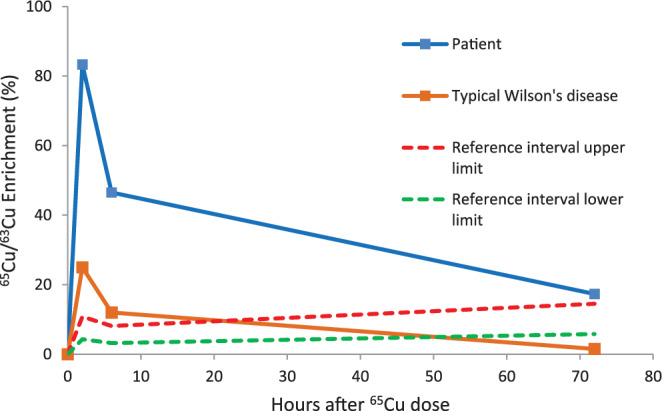
Graph showing copper‐65 absorption test results. Plasma ^65^Cu/^63^Cu enrichment was measured in samples collected at 0, 2, 6 and 72 hours relative to oral administration of a 3 mg ^65^Cu dose

Serum copper was low, 0.7 μmol/L (11‐22) with an undetectable CP of <0.02 g/L (0.20‐0.60). Urinary copper excretion was low‐normal at 0.17 μmol/d (<1) (Table 1). A copper‐65 absorption test was performed to investigate *in vivo* copper metabolism using a protocol based on that of Lyon et al.^4^ This test involves sequential analysis of plasma ^65^Cu/^63^Cu ratio by inductively coupled plasma mass spectrometry at time points up to 72 hours after administration of a 3 mg oral dose of the stable copper‐65 isotope. The results (Figure 1) showed a very pronounced early rise of plasma copper‐65 (83% enrichment at 2 hours post‐administration), followed by minimal incorporation of copper‐65 into the plasma protein pool between 6 and 72 hours post‐administration. This was consistent with an issue involving either production of the apo form of CP (such as occurs in ACP) or copper incorporation into apoCP to form holoCP.

**TABLE 1:** Key laboratory findings (pre‐treatment)

Analyte	Level	Reference interval
Ferritin (μg/L)	**1084**	12 to 250
Serum Iron (μmol/L)	**4**	14 to 31
Serum TIBC (μmol/L)	67	54 to 80
% Iron Saturation	**6**	20 to 55
Hb (g/L)	11.7	11.5 to 15
MCV (fL)	**68**	77 to 94
Caeruloplasmin (g/L)	**<0.02**	0.2 to 0.6
Serum Copper (μmol/L)	**0.9**	11 to 22
Urine Copper (μmol/day)	0.17	<1.00
ALT (U/L)	**87**	<50
HbA1c (mmol/mol)	**43**	20 to 41

*Note:* Abnormal parameters are marked in bold.

**FIGURE 2:**
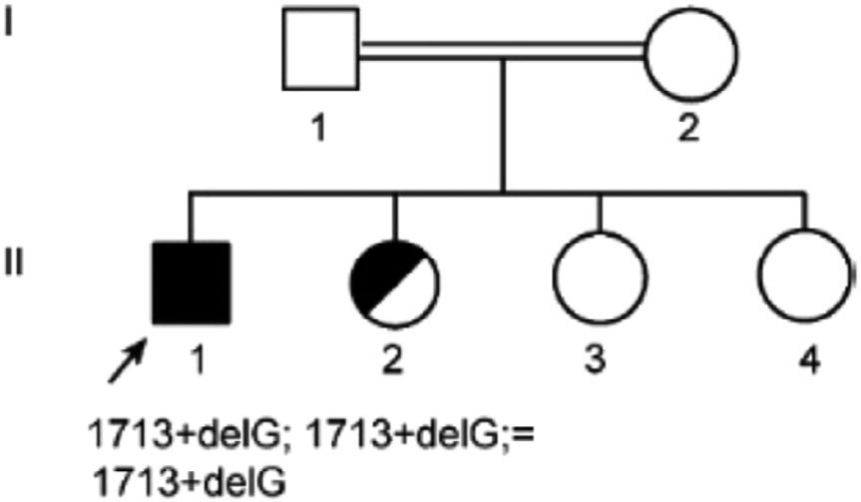
Family tree with marked index case

Subsequent next‐generation DNA sequencing identified that he was homozygous for a pathogenic splicing mutation c.(1713 + delG);(c.1713 + delG) in intron 9 of the *CP* gene. There was much consanguinity within the wider family (Figure 2); a cousin was also found to have been diagnosed with ACP and his maternal grandfather died from liver cancer.

**FIGURE 3:**
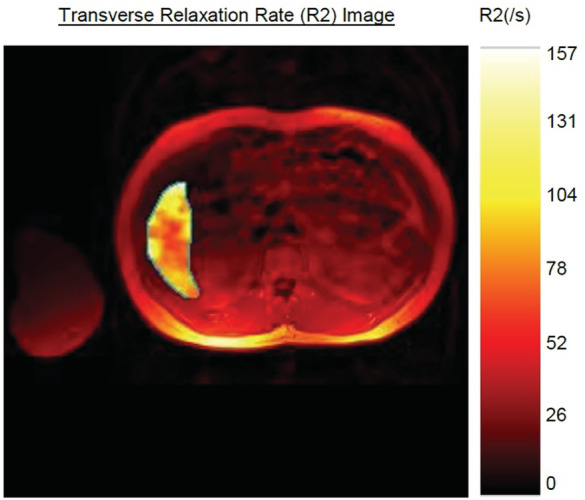
Pre‐treatment Ferriscan showing an elevated liver iron concentration of 94 mmol/kg dry tissue (reference range: 3‐33). Note the area of liver imaged for the Ferriscan excludes large vascular structures and other image artifacts

**FIGURE 4:**
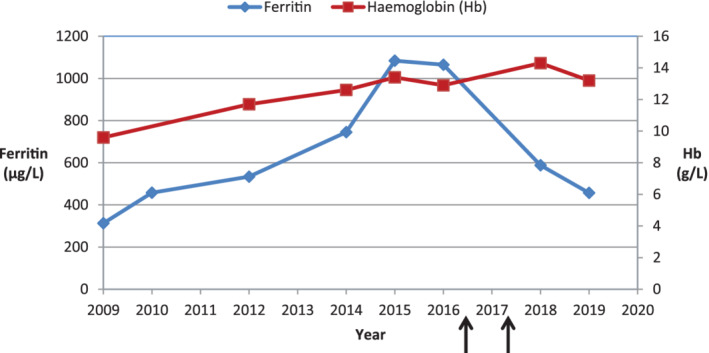
Graph showing ferritin and haemoglobin concentration over a 10‐year period, before and after commencing iron chelation therapy (first arrow from left, June 2016) and Octaplas (second arrow, April 2017)

At the age of 16, his height was 175 cm and weight 70.4 kg, giving a body mass index of 23 kg/m^2^ (77th percentile for age). At that time, assessment of liver iron deposition using Ferriscan identified an elevated liver iron concentration of 5.3 mg/g dry tissue (0.17‐1.8) (Figure 3). Magnetic resonance (MR) scanning of the brain did not identify any radiological evidence of iron deposition. Over a 7‐year period of biochemical monitoring, his ferritin gradually rose from 313 to 1084 μg/L (12‐250; Figure 4) together with a paradoxical iron deficient microcytic picture. Treatment with Octaplas, a solvent‐treated pooled human plasma, at the dose of 400 mL (2 units) was started with concurrent oral iron chelation therapy with deferasirox 540 mg daily (71 kg body weight). Octaplas was given monthly as an intravenous infusion.

Following a 2‐year treatment period, T2* liver MR imaging showed an average hepatic T2* value of 6.1 ms indicative of moderate liver iron loading. However, as this was a different modality to the pre‐treatment Ferriscan, we cannot comment on whether there had been a radiological improvement. Cardiac T2* MR imaging before and at 3‐year follow up did not detect any cardiac iron deposition. His neurological examination at 12‐month follow up was unremarkable.

His ferritin fell from 1084 (12‐250) to 457 μg/L (Table 1 and Figure 4). ALT reduced from 87 (<50) to 34 U/L. Haemoglobin rose from 11.9 g/L (11.5‐15) with an MCV of 68 fL (77‐94) to 14.3 g/L with an MCV of 74 fL, in keeping with an improvement in his apparent iron‐restricted erythropoiesis picture, prior to therapy. His marked leukonychia prior to treatment was noted to have resolved at recent clinic visits.

## 3 DISCUSSION

ACP was first described in 1987 in a patient found to have hypocaeruloplasminemia with biochemical and radiological evidence of iron accumulation.^5^ Accumulation of iron, not copper, was found in the liver and brain of the patient. Subsequently, earlier work was recognized demonstrating that although CP is the major copper‐carrying plasma protein, it is also a ferroxidase enzyme that is part of a complex regulatory system which protects the brain from both iron overload and deficiency. In ACP it is the loss of this regulation which underlies toxic iron accumulation in the brain (including the retina), as well as liver and pancreas.^6^ Indeed, the phenotype in these patients is dominated by iron overload, and not copper deficiency. Typically, neurological manifestations such as cognitive dysfunction and Parkinsonism appear in the fifth decade. MR imaging can be used to identify cerebral iron accumulation although the extent of this does not always correlate with the degree of neurological involvement.^6^ Iron‐restricted microcytic anaemia is recorded as the earliest manifestation of the disease occurring in 80% of those under the age of 20. Cardinal biochemical features include low serum iron, transferrin saturation, copper and caeruloplasmin, with raised ferritin. Unlike Wilson's disease, urinary copper levels are typically normal. Other common clinical manifestations seen include diabetes mellitus and retinal degeneration.^7^

The childhood‐onset of acaeruloplasminemia has not been well described, and we are not aware of any published cases of children diagnosed with this rare condition. The disease onset is often insidious and a diagnosis is made on investigation of characteristic symptoms. Our patient presented at the age of 12 with behavioral problems, concentration and memory lapses, leukonychia and bilateral lattice degeneration of the fundi increasing the risk of retinal degeneration. Prior to starting the combined treatment, he was confirmed to be a non‐diabetic. Regular monitoring of plasma glucose and HbA1c was performed and remained at non‐diabetic levels throughout, possibly adding further evidence of treatment efficacy.

The patient was found to be homozygous for a novel splicing mutation in intron 9, c.1713 + 1delG. This mutation alters the donor splice site of exon 9, which results in the skipping of this exon. His sister is a carrier of this splicing mutation (Figure 2) and his cousin is homozygous for the same mutation. There is little known about the pathogenicity of this mutation, however given his clinical phenotype, biochemical and radiological findings, it is expected to be a disease‐causing variant.

There is no widely accepted treatment regimen as most information has been gleaned from individual case reports to date. Currently, treatment is centered on reducing iron overload through the administration of iron chelators although case reports have described pooled plasma infusion, phlebotomy, oral zinc sulfate, minocycline and vitamin C or E administration. Although iron chelation appears effective in reducing systemic iron overload^8^, ^9^ there is less evidence of a beneficial effect in reversing or preventing the neurological manifestations which occur in ACP.^10^, ^11^ However, a recent analysis of aggregated case reports did conclude that neurological progression was slowed when iron chelation therapy was commenced early in the disease.^12^

Several case reports have described the use of the combination of iron chelation with caeruloplasmin replacement (FFP or stable equivalent such as Octaplas).^13^, ^14^, ^15^ Pooled plasma administration partially and temporarily restores CP levels, and serum iron levels rise after FFP infusion as a result of the ferroxidase activity of CP.^16^, ^17^ This tissue efflux of iron combined with iron chelation therapy to then remove it, is the mechanism by which this treatment approach is thought to work. With the half‐life of CP being 5.5 days^6^ the dosing interval of FFP has varied between case reports with the shortest interval being weekly.^14^ The decision was made to use Octaplas, and not FFP, as it appears to be safer (reduced risk of infections) in long‐term treatment. We opted initially for every two weeks however this was reduced to monthly due to poor venous access. There are no formal guidelines for Octaplas treatment in ACP, however biochemical monitoring of CP and ferritin levels post‐infusion may be helpful in setting a dose interval. ACP heterozygotes with CP levels of 0.08 to 0.1 g/L do not seem to manifest clinical symptoms in most cases^6^ suggesting this level is sufficient to maintain iron homeostasis and could be used as a target level for FFP treatment. Furthermore, CP in patients with Wilson's disease on long‐term chelation treatment with d‐penicillamine often falls to concentrations below the reporting limit of most immunoassays, 0.04 g/L, without any adverse effect on hematopoiesis (GTG: personal observation).

During the period of follow‐up, we did not encounter any safety issues with this combination therapy and the treatment was well tolerated. Plasma transfusion is not without risk; although rare these include transmission of infective pathogens, allergic reactions and transfusion‐related lung injury.^18^

## 4 CONCLUSION

This case report adds further evidence of treatment efficacy and safety in using combination therapy with FFP and iron chelation in ACP. We were able to demonstrate biochemical improvement in iron overload over a three‐year period with a reduction in ferritin and ALT, together with an improvement in his (iron‐restricted) microcytic anemia. However, owing to different liver imaging modalities before and after treatment, we were unable to quantify this radiologically. We did not encounter any adverse effects using this combination therapy. More studies are needed to assess treatment efficacy over a longer duration, with specific attention being paid to neurological outcomes. Moreover, establishing FFP dosing guidance, perhaps using CP level monitoring in order to titrate the FFP dose, would help to ensure optimal treatment at minimal risk to the patient and cost to the care provider.

## AUTHOR CONTRIBUTIONS

Andreas Tridimas—conception and design, drafting the manuscript, data extraction; Karolina M. Stepien, Andreas Tridimas, Godfrey T. Gillett, Adrian Williams, Sally Pollard, Nandini Sadasivam, Kirsty Mellor, Anthony Catchpole—conception and design, revising the manuscript critically for important intellectual content. Godfrey T. Gillett suggested initiation of plasma infusion therapy shortly after diagnosis. Anthony Catchpole performed the copper‐65 test and provided us with its interpretation. All authors read and approved the manuscript before submission.

## ETHICS STATEMENT

Written informed consent to publication has been obtained.

## GUARANTOR

AT and KMS.

## References

[1] Marchi G , Busti F , Lira Zidanes A , Castagna A , Girelli D . Aceruloplasminemia: A severe neurodegenerative disorder deserving an early diagnosis. Front Neurosci. 2019;13:325. 31024241 10.3389/fnins.2019.00325 PMC6460567

[2] Patel BN , Dunn RJ , David S . Alternative RNA splicing generates a glycosylphosphatidylinositol‐anchored form of ceruloplasmin in mammalian brain. J Biol Chem. 2000;275:4305‐4310. 10660599 10.1074/jbc.275.6.4305

[3] Pelucchi S , Mariani R , Ravasi G , Pelloni I , et al. Phenotypic heterogeneity in seven Italian cases of aceruloplasminemia. Parkinsonism Relat Disord. 2015;51:36‐42. 10.1016/j.parkreldis.2018.02.036 29503155

[4] Lyon TDB , Fell GSF , Gaffney D , McGaw BA , et al. Use of a stable copper isotope (^65^Cu) in the differential diagnosis of Wilson's disease. Clin Sci. 1995;88:727‐732. 10.1042/cs0880727 7634759

[5] Miyajima H , Nishimura Y , Mizoguchi K , et al. Familial apoceruloplasmin deficiency associated with blepharospasm and retinal degeneration. Neurology. 1987;37(5):761‐767. 3574673 10.1212/wnl.37.5.761

[6] Piperno A , Alessio M . Aceruloplasminemia: Waiting for an efficient therapy. Front Neurosci. 2018;12:903. 30568573 10.3389/fnins.2018.00903 PMC6290325

[7] Miyajima H , Hosoi Y . Aceruloplasminemia GeneReviews®. Seattle, WA: University of Washington; 2003. 20301666

[8] Tai M , Osamu M , Ichii T , Suzuki Y , et al. Case of presymptomatic aceruloplasminemia treated with deferasirox. Hepatol Res. 2014;44:1253‐1258. 24341521 10.1111/hepr.12292

[9] Roberti MDRF , Filho HMB , Gonçalves CH , Lima FL . Acerulopaslamiaemia: a rare disease – diagnosis and treatment of two cases. Rev Bras Hematol Hemoter. 2011;33:389‐392. 23049345 10.5581/1516-8484.20110104 PMC3415789

[10] Kono S . Aceruloplasminemia: an update. Int Rev Neurobiol. 2013;110:125‐151. 24209437 10.1016/B978-0-12-410502-7.00007-7

[11] Dusek P , Schneider SA , Aaseth J . Iron chelation in the treatment of neurodegenerative diseases. J Trace Elem Med Biol. 2016;38:81‐92. 27033472 10.1016/j.jtemb.2016.03.010

[12] Vroegindeweij LHP , Boon AJW , Wilson JHP , Langendonk JG . Effects of iron chelation therapy on the clinical course of aceruloplasminemia: an analysis of aggregated case reports. Orphanet J Rare Dis. 2020;15:105. 32334607 10.1186/s13023-020-01385-w PMC7183696

[13] Logan JI , Harveyson KB , Wisdom GB , Hughes AE , Archbold GPR . Hereditary caeruloplasmin deficiency, dementia and diabetes mellitus. QJM. 1994;87:663‐670. 7820540

[14] Poli L , Alberici A , Buzzi P , et al. Is aceruloplasminemia treatable? Combining iron chelation and fresh‐frozen plasma treatment. Neurol Sci. 2017;38(2):357‐360. 27817091 10.1007/s10072-016-2756-x

[15] Yonekawa M , Okabe T , Asamoto Y , Ohta M . A case of hereditary ceruloplasmin deficiency with iron deposition in the brain associated with chorea, dementia, diabetes mellitus and retinal pigmentation: administration of fresh‐frozen human plasma. Eur Neurol. 1999;42(3):157‐162. 10529542 10.1159/000008091

[16] Lee GR , Nacht S , Christensen D , Hansen SP , Cartwright GE . The contribution of citrate to the ferroxidase activity of serum. Proc Soc Exp Biol Med. 1969;131(3):918‐923. 5791811 10.3181/00379727-131-34009

[17] Fieden E , Hsieh HS . The biological role of ceruloplasmin and its oxidase activity. Adv in Exp Med Biol. 1976;74:505‐509. 183481 10.1007/978-1-4684-3270-1_43

[18] Pandey S , Vyas GN . Adverse effects of plasma transfusion. Transfusion. 2012;52(suppl 1):65S‐79S. 22578374 10.1111/j.1537-2995.2012.03663.x PMC3356109

